# Use of Whole-Genome Sequencing for Food Safety and Public Health in the United States

**DOI:** 10.1089/fpd.2019.2662

**Published:** 2019-07-09

**Authors:** Eric Brown, Uday Dessai, Sherri McGarry, Peter Gerner-Smidt

**Affiliations:** ^1^ Center for Food Safety and Applied Nutrition, Food and Drug Administration, College Park, Maryland.; ^2^ Office of Public Health Science, Food Safety and Inspection Service, United States Department of Agriculture, Washington, District of Columbia.; ^3^ Enteric Diseases Laboratory Branch, Centers for Disease Control & Prevention, Atlanta, Georgia.

**Keywords:** whole-genome sequencing, WGS, surveillance, food safety, FDA, FSIS, CDC

## Abstract

Whole-genome sequencing (WGS) is increasingly used by food regulatory and public health agencies in the United States to facilitate the detection, investigation, and control of foodborne bacterial outbreaks, and food regulatory and other activities in support of food safety. WGS has added a level of precision to the surveillance leading to faster and more efficient decision making in the preparedness and response to foodborne infections. In this review, we report the history of WGS technology at the Centers for Disease Control & Prevention (CDC), the Food and Drug Administration (FDA), and the United States Department of Agriculture's Food Safety and Inspection Service (USDA/FSIS) as it applies to food safety. The basic principle of the method, the analysis, and interpretation of the data are explained as is its major strengths and limitations. We also describe the benefits and possibilities of the WGS technology to the food industry throughout the farm-to-fork continuum and the prospects of metagenomic sequencing applied directly to the sample specimen with or without pre-enrichment culture.

## Introduction

The United States has a diverse food supply with a significant amount of food that is imported from other countries. Ensuring the safety of both domestically produced and imported foods is a daunting task.

Federal and state agencies, including the U.S. Department of Health and Human Services' agencies, Centers for Disease Control & Prevention (CDC), and the Food and Drug Administration (FDA), and the United States Department of Agriculture's Food Safety and Inspection Service (USDA/FSIS) must collaborate to ensure safety measures are met to protect the American people. Scientific advances have enabled us to make continuous progress in making our foods safer by accurately identifying, controlling, and preventing microbial and chemical hazards in foods. For instance, the introduction of pulsed-field gel electrophoresis (PFGE) in PulseNet, the nation's molecular subtyping network for surveillance of foodborne infections (Swaminathan *et al.*, 2001), revolutionized the detection, investigation, and control of outbreaks over the past two decades.

While PFGE served well for the intended purpose, it has limitations for molecular characterization and subtyping of bacterial pathogens, in particular suboptimal precision. Recent developments in our ability to sequence an entire bacterial genome in a timely and cost-efficient manner have brought us closer to this goal by providing us with information about a pathogen at the DNA and gene level with previously unheard-of precision (Aarestrup *et al.*, 2012; Allard *et al.*, 2018). The process for doing this is broadly called whole-genome sequencing (WGS).

The original sequencing technology, called Sanger sequencing (Sanger *et al.*, 1977), was a breakthrough method for highly accurate sequencing of relatively short DNA fragments (a few 1000 bp). For sequencing longer stretches of DNA, the process was time consuming and involved multiple reactions. The human genome and the first bacterial genome were sequenced using the Sanger technology and it took several years and cost millions of dollars (Fleischmann *et al.*, 1995; Venter *et al.*, 2001).

The introduction of next-generation sequencing (NGS) in the early 2000s revolutionized the way DNA sequencing could be applied to food safety and public health on a routine basis. With the NGS approach, an entire bacterial genome can be sequenced in small random fragments (<100 to several 1000 bp) multiple times in a single reaction (a technique called “massive parallel sequencing”), after which the full DNA sequence is determined electronically by connecting fragments with overlapping sequences, using sequence assembly software (Margulies *et al.*, 2005; Vincent *et al.*, 2017). With the advent of NGS, it became possible to sequence whole genomes in a matter of days at a cost of a few 100 dollars.

The technology is constantly improving and becoming both faster and cheaper. Concurrent investments and developments in other fields such as microbial ecology, evolutionary biology, epidemiology, bioinformatics, and information technology has transformed our ability to use genomic sequence information to enhance food safety and public health (Allard *et al.*, 2018). Specifically, the widespread availability of small, easy–to-use next-generation sequencers is causing a paradigm shift in the way scientists identify pathogens and their sources.

In addition, WGS analysis of microbial pathogens is now supplanting and replacing many traditional microbiological analyses to identify and characterize bacteria, for example, serotyping, antimicrobial resistance (AMR), and virulence profiling in a single WGS workflow that is rapid and cost efficient (Allard *et al.*, 2016; Carleton and Gerner-Smidt, 2016). Thus, this technology is ideally suited for use in national and international surveillance systems in support of food safety and public health. Apart from improving outbreak detection, and response, it will likely revolutionize microbiological source attribution of sporadic foodborne illness and expand our knowledge of the epidemiology of different infectious diseases in the years to come.

In this review, we describe how the food regulatory agencies (FDA and FSIS) and their public health partners in the states and local jurisdictions, together with the CDC, use WGS to support their mission of protecting Americans from illness caused by foodborne bacterial pathogens.

## History of WGS at U.S. Food Safety Federal Agencies, Including Present Time

The FDA's Center for Food Safety and Applied Nutrition (CFSAN) established in 2013 the GenomeTrakr WGS Network (Allard *et al.*, 2016), the first integrated network of state and federal laboratories to use WGS to track foodborne pathogens to improve outbreak response activities related to FDA compliance and regulatory programs by providing more precise scientific traceback and environmental source data (https://www.fda.gov/food/foodscienceresearch/wholegenomesequencingprogramwgs) (Allard *et al.*, 2012; Hoffmann *et al.*, 2016; Stevens *et al.*, 2017). The network has created a publicly available global database containing the genetic makeup of thousands of foodborne disease-causing bacteria from food and environmental sources housed at the National Center for Biotechnology Information (NCBI) at the National Institutes of Health (NIH). The FDA works closely with NCBI to develop publicly available analytical software tools associated with the GenomeTrakr database (https://www.ncbi.nlm.nih.gov/pathogens*).*

Participants in GenomeTrakr include 14 federal laboratories (including USDA-FSIS food laboratories), and state agriculture, food, environmental, and public health laboratories in 14 states, 1 U.S. hospital laboratory, and 9 international laboratories (including laboratories from Mexico, Argentina [through the World Health Organization, WHO], and England). Other partners include the PulseNet USA network, reference laboratories in Ireland, Denmark, Canada, and a number of other countries (https://www.fda.gov/food/whole-genome-sequencing-wgs-program/genometrakr-network). The goal of the network is to further enhance the GenomeTrakr database and network by adding partners from public health and regulatory agencies around the world.

Currently, the FDA sequences all *Salmonella*, Shiga toxin-producing *Escherichia coli* (STEC), and *Listeria monocytogenes* isolates as they are received when isolated as part of its investigations into foodborne contamination events, as well as to support its compliance and sampling programs (Allard *et al.*, 2016; Stevens *et al.*, 2017).

The CDC used WGS for the first time to characterize an enteric pathogen in 2010, as part of the newly discovered cholera outbreak in Haiti. This way, the investigators confirmed the hypothesis of a single-source introduction with a variant of a strain that had its genetic origin in South Asia. At the time, CDC did not have bioinformatics capacity to analyze the sequences, so it partnered with the Public Health Agency of Canada (Reimer *et al.*, 2011). The CDC shared the sequences of three outbreak isolates in the public domain of the Sequence Read Archive at NCBI.

Due to this successful application of the technology and a series of several successful retrospective WGS studies on outbreaks performed by FDA and others (Gilmour *et al.*, 2010; Lewis *et al.*, 2010; Lienau *et al.*, 2011; Aarestrup *et al.*, 2012; Allard *et al.*, 2012), the regulatory and public health agencies decided to assess the utility of WGS as a tool to supplement or even replace PFGE as the preferred subtyping method for use in PulseNet. Initially, the technology was applied to retrospective characterization of historical isolates of different species to assess its potential for improving outbreak investigations.

However, while these retrospective studies helped affirm the proof of principle of the technology, it could not prove that WGS would work for routine real-time surveillance. For that reason, in 2013, the PulseNet central laboratory at the CDC together with its partners at FDA, FSIS, and NCBI, and state laboratories participating in GenomeTrakr and PulseNet, launched a pilot project for sequencing and analyzing isolates of *L. monocytogenes* in real time, in parallel with the current PFGE-based surveillance (Jackson *et al.*, 2016). *Listeria* was chosen because the organism is foodborne, causes serious illness with several outbreaks occurring each year, and its genome is fairly small (Jackson *et al.*, 2016). In addition, the associated surveillance of *L. monocytogenes* by the food regulators and epidemiologists is well established.

This pilot study demonstrated that application of WGS to laboratory surveillance provides higher resolution and precision than PFGE, and as a result more outbreaks could be detected, investigated, and controlled than ever before, and conversely false outbreak signals by PFGE could be debunked by WGS. The pilot study also showed that very small outbreaks (e.g., with down to two clinical cases) could be detected through matching the sequences of the patient isolates to food isolates already sequenced by FDA or FSIS (i.e., “retrospective” surveillance, see section on Retrospective Outbreak Investigations).

With funding from CDC's Advanced Molecular Detection initiative, PulseNet has worked toward implementing WGS into routine public health surveillance since 2014. This change was implemented for surveillance of *Listeria* and *Campylobacter* in 2018, with STEC and *Salmonella* in 2019 (Ribot *et al.*, 2019; Tolar *et al.*, 2019).

The FSIS works closely with the Agricultural Research Service (USDA-ARS), CDC, and its public health partners to make improvements in its hazard detection and characterization methods. As WGS technology was evolving, FSIS initially partnered with FDA, CDC, and NCBI, to contribute *L. monocytogenes* isolates from food sources to the interagency WGS project. The earliest FSIS isolates that were subject to WGS were *L. monocytogenes* isolates. These were sequenced by the FDA as a part of the interagency *L. monocytogenes* project.

Early on, FDA also sequenced outbreak-related *Salmonella* for FSIS. In 2014, FSIS collaborated with its partners to establish an *in-house* WGS capability and for the first time sequenced *Salmonella* and *L. monocytogenes*, and subsequently added STEC and *Campylobacter* in 2015. When FSIS started WGS in its laboratories, the initial focus was to sequence isolates of food pathogens that were associated with outbreaks. For instance, in 2014, FSIS conducted WGS on *Salmonella* Heidelberg associated with a large outbreak associated with chicken (https://www.fsis.usda.gov/wps/portal/fsis/topics/recalls-and-public-health-alerts/recall-case-archive/archive/2014/recall-044-2014-release) and in 2015 with *Salmonella* I 4,[5],12:i:- that was associated with pork (https://www.fsis.usda.gov/wps/portal/fsis/topics/recalls-and-public-health-alerts/recall-case-archive/archive/2015/recall-110-2015-release-expansion).

From 2016, FSIS continually expanded sequencing capacity and began sequencing all the pathogens isolated in its routine sampling. From 2016, FSIS also started sequencing *Salmonella* and *Campylobacter* isolated from animal cecal samples from the National Antimicrobial Resistance Monitoring System (NARMS) surveillance program. FSIS uploads all the sequences and associated metadata to NCBI in real time. In collaboration with it public health partners, FSIS uses information from both WGS and PFGE in its regulatory decision-making process when applicable.

## Analytical Methods

There are two analytical postsequencing approaches to make raw nucleotide data of an entire sequence of a bacterial genome understandable to compare the relationship between two bacterial isolates: base by base (single-nucleotide polymorphism [SNP] analysis) or gene to gene (multilocus sequence typing [MLST]). Both approaches can be used to link clinical isolates back to environmental or contaminated food sources, or to cluster food and environmental isolates across time and space for the detection of outbreaks. These fundamentally different, but complementary analytical approaches are used routinely by the federal partners to meet their investigative or regulatory needs.

For the first approach, an SNP is a nucleotide difference at a specific position in the genome of a test strain relative to the sequence of a reference strain and occurs because of a genetic mutation event.

SNPs occur throughout the genome, including both in coding regions (genes) and noncoding regions. The choice of a reference genome for SNP identification (“SNP calling”) is important and is tailored to any given situation (e.g., a reference closely related to an outbreak strain provides the most accurate assessment of the SNP differences in an outbreak setting). DNA stretches that are only present in the reference or in the test isolates are ignored in the analysis. Once the SNPs have been called in all the test isolates, the SNP profiles of all isolates are compared in a pairwise manner and usually displayed in the form of a phylogenetic tree. SNPs present on mobile elements (e.g., phages, indels [insertions or deletions], and plasmids) may not be phylogenetically relevant. They are therefore often filtered out in the final analysis to remove noise from the epidemiological investigation.

SNP analysis uses almost all the genetic information from a genome/strain, and thus provides the theoretically highest level of precision available for the reconstruction of strain phylogeny, hence the term high-quality SNP analysis. The SNP approach is the primary tool used by FDA-CFSAN, USDA-FSIS, and many other GenomeTrakr partners. Through an *in-house* process, FDA subjects SNPs and analysis thereof to rigorous quality checks to ensure reliability, stability, and authenticity before this information is made available in the public domain (https://github.com/CFSAN-Biostatistics/snp-pipeline) (Pettengill *et al.*, 2014).

The MLST approach works by assessing sequence variations in the coding regions of the genes (or “loci”) (Maiden *et al.*, 2013). Any differences (i.e., SNPs, indels and recombinations) are assessed. This approach is very flexible because the number and the nature of the genes assessed can be tailored to any given situation and genomes in question. PulseNet currently applies three levels of discrimination: (1) seven housekeeping gene (7-gene) MLST, (2) core genome (cg) MLST that assesses genes present in almost all strains of a given species, and (3) whole genome (wg) MLST that assesses almost all genes present in any strain in a given species.

The 7-gene MLST approach allows the splitting of isolates of a species in a broad phylogenetic context, called the sequence types and clonal complexes. The cgMLST provides the most detailed phylogenetically relevant differentiation of a species. The wgMLST provides even more discrimination than cgMLST, and is thus useful for cluster investigations to discriminate between closely related isolates, although observed differences may not reflect true phylogeny since the accessory genome (e.g., genes not present in all isolates) is also analyzed.

The genes identified in test strains are compared against a reference database of genes with all known gene variants (“alleles”) of the species/pathogen assessed. The reference in MLST is therefore a database of loci and alleles from numerous strains and not just a single reference genome. Each unique allele sequence is given a number and genomes are compared based on the allele numbers, similar to the way SNP profiles are being compared. Like with SNP analysis, mobile elements are often filtered out in the final analysis with wgMLST because the differences observed may not always be epidemiologically relevant. In contrast to SNP analysis, MLST cannot assess variations in noncoding regions (i.e., between genes).

MLST is the primary analytical approach used by PulseNet. A validated database with a shared nomenclature is used for comparisons. The use of publicly shared databases is recommended by PulseNet International (Nadon *et al.*, 2017). The cgMLST schemes used by PulseNet are either identical to existing publicly available schemas (e.g., the *L. monocytogenes* schema developed by Institut Pasteur, France [http://bigsdb.pasteur.fr/listeria] and the *Campylobacter* schema from the University of Oxford, United Kingdom [https://pubmlst.org/campylobacter], or inspired by, and overlapping with public schemas [e.g., the *Salmonella* and *E. coli* schemas from University of Warwick, United Kingdom; https://enterobase.warwick.ac.uk/species/index/senterica and https://enterobase.warwick.ac.uk/species/index/ecoli]). MLST allele databases must be curated to ensure their quality, and at this time, most curation is automated. Manual curation in PulseNet is the responsibility of subject matter experts at CDC and its international partners.

Theoretically, wgMLST analysis is not as discriminatory as SNP analysis because the latter assesses intergenic regions at variance with the former; additionally, the ability to choose closely related reference strain(s) for the precise assessment of the relatedness of genetically similar isolates in the SNP approach helps resolve certain relatedness calls where wgMLST could fail. However, for practical purposes, the approaches appear to be equally discriminatory and epidemiologically concordant (Chen *et al.*, 2017; Cunningham *et al.*, 2017; Katz *et al.*, 2017).

Another important practical difference between the SNP and MLST approaches relates to the level of computational support that is required. Open-source command-line software, which is typically used for SNP analysis, usually runs on a Linux computer in a high-performance infrastructure by bioinformaticians. The 7-gene/cg/wg MLST analysis on the other hand is often run on a Windows-based platform using commercial software. Such commercial “off-the-shelf” software may be used by scientists with less bioinformatics skills. The latest iterations of several commercial softwares available for MLST analysis also include SNP-pipeline functionality (e.g., most recent versions of Bionumerics (Applied Maths, Austin, TX) and the shareware GalaxyTrakr (https://www.galaxytrakr.org), which contains the FDA SNP pipeline).

Since the two approaches assess genetic variants in a different way, they both should be used when one of them does not provide clear-cut answers or when stronger support for an association needs to be explored.

Ultimately, the choice of the primary analytical approach depends on the needs of the end user. Since PulseNet participants are microbiologists and not bioinformaticians, and rely on both the decentralized generation and analysis of data in a central database, only a fully standardized scheme that may be used to subtype all members of a given pathogen/species will work in the network. MLST has these qualities and is therefore the primary analytical approach used in the network. SNP analysis is used by PulseNet to confirm the similarity of isolate sequences as determined by MLST in more complex outbreaks and when MLST provides inconclusive results.

On the other hand, FDA preferentially applies an SNP-based approach; this method is currently more fit for the purpose of identifying products associated with an outbreak. FSIS partners with the CDC on foodborne outbreak investigations and with the FDA on pathogen investigations, especially with *L. monocytogenes* in dual jurisdiction establishments. As a result, FSIS has adopted both SNP and MLST approaches for its pathogen pipelines.

The CDC, FDA, FSIS, and the other public health partners make their sequence data available to the food safety community by uploading them to the public domain at NCBI in near real time. The number of uploads by the agencies and the domestic GenomeTrakr and PulseNet networks until May 1, 2019, is provided in Table 1.With every WGS upload, a limited sample of metadata is also shared in NCBI. The NCBI Pathogen Detection website (https://www.ncbi.nlm.nih.gov/pathogens) provides daily new SNP-based phylogenetic trees for all publicly available data. Users only need to upload their draft genomes and then search for their results the following day using online web browsing tools at the site.

**Table 1.** Number of Uploads^1^ of Raw Sequences of *Salmonella, E. coli*^2^, *Campylobacter*, *Listeria*, and Other Foodborne Bacterial Pathogens by Network or Agency to the GenomeTrakr Databases at NCBI.

	*PulseNet/CDC*	*GenomeTrakr/FDA*	*FSIS*	*Total*
*Salmonella*	78,411	41,503	17,219	137,133
*E. coli*	25,304	3,637	2,466	31,407
*Campylobacter*	9,497	2,666	11,804	23,967
*Listeria*	5,047	12,365	702	18,114
Other^3^	608	825	978	2,411
Total	118,867	60,996	33,169	213,032

1 : the number includes all sequences uploaded until May1, 2019

2 : *E. coli* includes STEC, other pathotypes, *Shigella* and commensal *E.coli*

3 : other foodborne pathogens include *Yersinia, Cronobacter, Clostridium* and *Vibrio spp.*

CDC, Centers for Disease Control &Prevention; FDA, Food and Drug Administration; FSIS, Food Safety and Inspection Service; NCBI, National Center for Biotechnology Information.

## Use of WGS in Routine Inspection Process

Although the U.S. food safety and public health agencies differ in their regulatory constructs and jurisdictional responsibilities, inspection and verification of food safety systems remain an integral part of their mandate. While the initial application of WGS has been to investigate and resolve foodborne outbreaks, the unprecedented ability of WGS to provide detailed characteristics of pathogenic isolates can be seamlessly integrated into routine inspection and verification processes. An effective integration of WGS analytics in the inspection and verification processes can enable federal partners to understand issues related to pathogen introduction, harborage, cross contamination, source attribution, temporal and geographic distribution, industry-specific trends, and other attributes.

Recognizing the potential of WGS, FSIS committed to implementing WGS as a part of its inspection modernization efforts, and in 2016, the agency published its intent to use WGS information in its inspection process (FSIS, 2016). To inform the public and industry about the use of WGS in regulatory settings, FSIS and the federal public health agencies, in collaboration with other national and international experts, held a public meeting in October 2017 (FSIS, 2017). For enhancing the sanitary practices and programs in the regulated establishments, FSIS is committed to using WGS analytics in the development of individualized inspection strategies for certain food pathogens. Use of WGS in routine inspection and verification helps FSIS and public health partners to detect and prevent contamination events and ensuing foodborne illness outbreaks.

## AMR and Mobile Elements

WGS also enables an assessment of the genes that are responsible for AMR, their location (main genome or mobile elements), as well as the potential for multidrug resistance and rapid dissemination. The CDC, FDA, FSIS, and ARS have actively collaborated on nationwide AMR surveillance for the past 20 years in the NARMS program (Karp *et al.*, 2017). Until recently, this surveillance was primarily based on the phenotypic assays for the detection of AMR. However, from 2015, the NARMS program started sequencing all *Salmonella* in foods and an increasing number of clinical isolates (McDermott *et al.*, 2016); in 2016, all *Campylobacter* isolates were added. Application of WGS to NARMS enabled the program to detect the introduction and spread of novel AMR genes in the United States from any source monitored.

Recent examples include the extended-β-lactamase CTX-M-65 gene in *Salmonella* serotype Infantis that were isolated from food animals and retail chicken, and the identification of plasmid-mediated quinolone resistance in *Salmonella* that were isolated from swine intestines/ceca and retail pork chops (Tate *et al.*, 2017; Tyson *et al.*, 2017). To prevent the potential for AMR spread and consumer exposure through food products, the FSIS and NARMS partners promptly share findings of concern for immediate action. The ability to perform WGS and access sequence information as part of public domain are the primary reasons that the NARMS program was able to detect plasmid-mediated CTX-M-65 and quinolone resistance genes in the United States in a timely manner.

## Data Interpretation

**FIG. 1.**
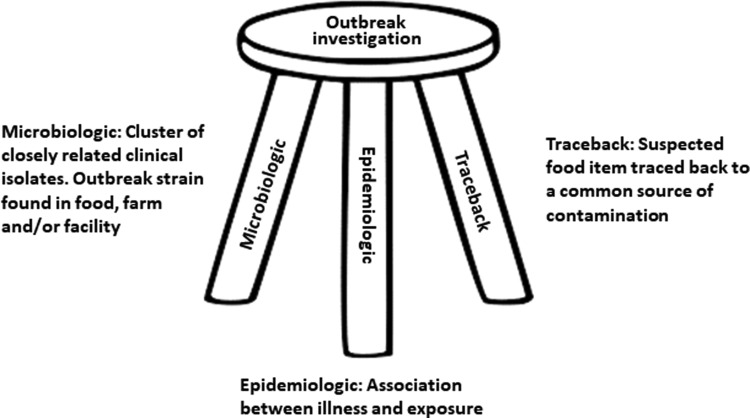
The three-legged stool of outbreak investigation.

It should be kept in mind that the microbiological data can never stand alone and must be interpreted in the context it is going to be used. A simplified illustration of the core elements in an outbreak investigation is shown in Figure 1. The biological basis of interpretation of sequence data is straightforward. Isolates that are highly similar share a recent common ancestor and conversely less similar isolates are less likely to share a recent common ancestor. However, biology does not always correlate with epidemiology 100%, even though it is a fundamental assumption in molecular epidemiology that the biological similarity also reflects epidemiological relatedness.

**FIG. 2.**
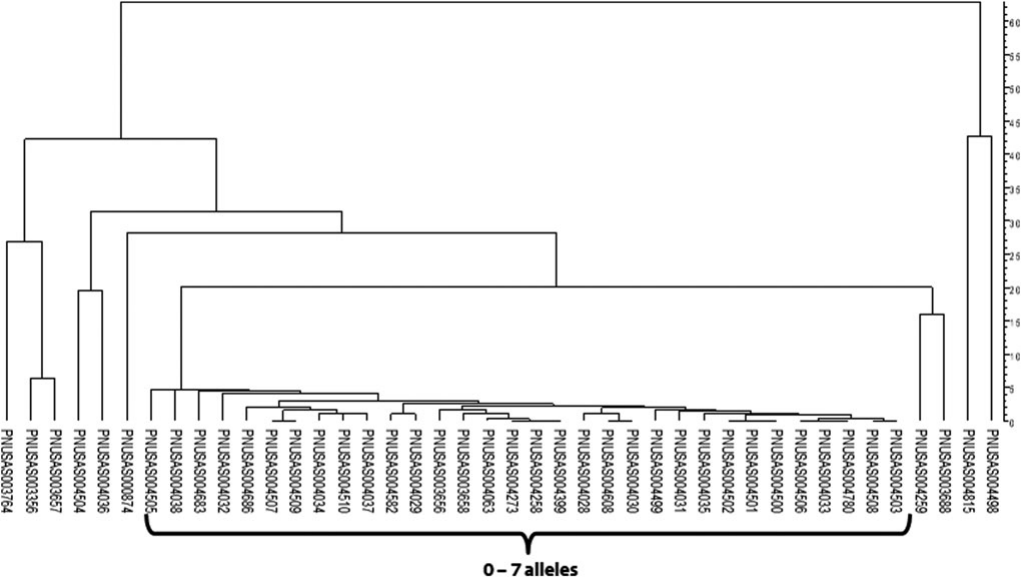
WGS data are contiguous. An outbreak strain is typically defined by a small range of SNPs/alleles, in this case, a *Salmonella* serotype Newport outbreak with isolates differing from each other by up to seven alleles.

It is important to note that since foodborne pathogens retain markedly short generation times under standard growth conditions, a small number of genetic changes (i.e., in SNPs or allele differences), will accrue over time, including from the time of product contamination to the isolation of the clinical specimen or subsequent resampling of a facility or product. Therefore, it is likely that a small, but distinct difference will be observed with regard to the number of SNPs or allele differences among clinical, food, animal, and environmental isolates, despite all of them being part of the same outbreak. Although seen a few SNPs/alleles apart, these strains will cluster together distinct from unrelated isolates (Fig. 2).

When molecular linkages are made based on closely related WGS profiles, scientists conduct a detailed analysis of their relatedness in the light of associated metadata, including, but not limited to, time, sources, and spatial distribution. The stable SNP/allele signatures can be mapped onto a phylogenetic tree and serve as biomarkers for particular strains or groups of strains throughout an investigation. The detailed *in-house* SNP analysis conducted at the FDA is important, as these WGS data could comprise a portion of the physical evidence, should related regulatory action ensue following an investigation into a food contamination event.

When interpreting sequencing data, it is important to consider whether this biological result makes epidemiological sense. It is impossible to define a single cutoff of SNP/allele differences that defines “closely related,” which fits all situations (e.g., any type of outbreak); since the clonality of different species and even serotypes vary, some epidemiological contexts allow more evolution (i.e., increasing biological variation) than others, and no two data analysis pipelines will measure exactly the same differences.

For this reason, U.S. federal agencies consider cutoffs as rough guidelines that are used along with other information such as, the clonality of an organism, its diversity in the farm-to-fork continuum, the epidemiological question to be answered, and the analytical pipeline used. With this in mind, federal agencies differentiate between three scenarios: (1) situations where isolates are closely related, (2) situations where isolates are not related, and (3) a scenario where isolates are neither closely related nor unrelated.

In the first situation, the isolates being compared may typically differ from each other by 0–20 SNPs/alleles. Although biologically they most likely share a recent common ancestor and epidemiologically they may likely share a common source in the production and distribution chain, additional information related to all aspects of the product, processing, and distribution is required as a part of the totality of evidence to make any connection. This is the typical scenario that is encountered with point-source outbreaks (Taylor *et al.*, 2015; Wilson *et al.*, 2016; Chen *et al.*, 2017; Crowe *et al.*, 2017a). When highly related isolates are found in different zones in a food production facility, it is most likely that a single strain has spread within that facility. Additional sampling and investigation may be needed to establish the transmission chain; WGS can be an important tool to elucidate and target the origins of identified contamination and thus help mitigate the problem.

Sometimes an epidemiological link between genetically indistinguishable isolates is not immediately apparent. Stasiewicz *et al.* found indistinguishable *L. monocytogenes* isolates in deli stores across different states in the United States, but did not find any apparent link between the stores (Stasiewicz *et al.*, 2015). The FSIS continues to encounter similar situations when a WGS match cannot be supported by information that can link regulated products to the patients. Performing tracebacks is often a very complex task and isolates might be linked to each other at an unidentified earlier point or place in the production and distribution chain. Thus, a genetic “match” is a hypothesis of shared ancestry, but by itself cannot be sufficient for conclusive regulatory actions. A WGS match is one signal that always is combined with additional information, such as a firm's compliance history, and may warrant follow-up activities, including possible microbiological examination.

Thus, the FDA or FSIS does not automatically proceed with any type of regulatory action solely based on WGS findings, but uses all investigative information and inspectional findings as part of their decision-making process. However, the presence of adulterant pathogens in foods or in those parts of a facility that come into contact with finished products is nevertheless a public health concern, and its mitigation would not necessarily rest on whether or not associated human illnesses are reported.

Isolates that differ a lot from each other, typically by >50–100 SNPs/alleles, often do not share the same source. However, the presence of multiple strains on a food production farm or in a facility could indicate insanitary conditions that should be addressed immediately. It is not uncommon to see polyclonal outbreaks, with multiple pathogenic strains causing an outbreak associated with a single food source.

A recent example is the *Salmonella* outbreak associated with consumption of raw tuna scrape imported from India (https://www.cdc.gov/salmonella/bareilly-04-12/index.html) that involved two serotypes, Bareilly and Nchanga. Other examples include a *Salmonella* outbreak associated with consumption of chicken from the same producer (https://www.cdc.gov/salmonella/heidelberg-10-13/index.html) and an *L. monocytogenes* outbreak related to consumption of contaminated ice cream (Chen *et al.*, 2016).

The situations where isolates are not clearly closely related, but not genetically unrelated either, typically with SNP/allele differences in the range between 20 and 50, are difficult to interpret. Such differences are often seen in zoonotic outbreaks (i.e., outbreaks caused by contact with animals where the outbreak strain constantly evolves during *in vivo* propagation in the reservoir).

A recent outbreak (https://www.cdc.gov/salmonella/small-turtles-03-12/epi.html) associated with exposure to live small turtles illustrates this situation further. In this case, three *Salmonella* serotypes were involved: Poona, Pomona, and Sandiego, and the serotype Poona differed by up to 17 SNPs from each other and serotype Pomona isolates by up to 30 SNPs. This situation may also be observed in foodborne outbreaks. In the *Salmonella* serotype Heidelberg outbreak, some of the outbreak strains as defined by PFGE showed up to 22 SNP differences (Crowe *et al.*, 2017b). This outbreak occurred over a period of several years, likely enabling *in vivo* propagation of the variants of the same strain/genotype in chicken production. In the *L. monocytogenes* ice cream outbreak, two of the outbreak strains differed by up to 29 SNPs (Chen *et al.*, 2017) or 25 alleles (CDC, unpublished). In this outbreak, the outbreak strain also likely persisted in the production environment for many years, thereby enabling its growth and diversification.

## Retrospective Outbreak Investigations

A unique advantage of using WGS in an integrated manner is that outbreaks may be identified at an earlier stage and therefore resolved faster. For instance, in the case of *L. monocytogenes,* the average size of outbreaks since the application of WGS has become smaller with more outbreaks being solved, and solved faster than in the past (Jackson *et al.*, 2016). With early intervention and timely response of regulators and industry, outbreaks may be controlled before they spread.

The decrease in the median number of case patients in outbreaks is, to a large extent, driven by retrospective’ or source-driven outbreak investigations. In the past, clusters of isolates from the food supply, “matching” a few clinical isolates, were seldom followed up epidemiologically because most signals were false because of the poor resolution of PFGE. This has changed with the implementation of WGS. With that method, a “match” between food or food production environmental isolates and a few clinical isolates has a much higher chance of reflecting an epidemiological connection because the extremely high precision of WGS. Therefore, more such clusters are investigated and the connection confirmed epidemiologically. The *Listeria* outbreak associated with consumption of stone fruits in 2014 with just two confirmed cases is an example of a typical retrospective outbreak investigation (Jackson *et al.*, 2015).

However, these retrospective outbreak investigations will likely not become the new norm to solve all outbreaks. With PFGE in 2016, ∼14% of clusters of clinical isolates of *Salmonella*, STEC, and *L. monocytogenes* included a nonhuman isolate at the time of detection (PulseNet, unpublished). It is unclear, at this time, how WGS will change this. On one hand, sequencing has much a higher precision than PFGE, meaning that fewer illness clusters will contain nonclinical isolates at the time of detection. On the other hand, intensified and more targeted sampling of the food and production environment will logically have the opposite effect.

## Future Prospects

### Food industry benefits

WGS provides information beyond the identity and relationship of strains; it can also help public health by improving the safety, quality, and shelf life of foods. For example, WGS can provide information about pathogenicity and virulence, adaptation and survival, resistance to biocides, metals, and antimicrobial drugs, and the plasticity of genomes. This is valuable for regulators and industry alike to help design, prioritize, and implement interventions to prevent and contain strains of significant public health concern from entering the U.S. food supply. In addition, this information will also help develop metagenomic approaches (direct sequencing of all genetic material in a sample) to understand the microbial communities and the genes of interest directly from a food sample without the need for isolating pathogenic or other bacteria.

The food production industry has just started to use WGS findings within a facility to resolve the issue of transient (WGS profiles unique or unrelated) and resident (WGS profiles closely related) pathogens as powerful tracking tools to trace sources of contamination, as part of environmental monitoring requirements. A few companies are already employing WGS to resolve contaminated niche locations within their production lines, which helps prevent their finished products from ever becoming contaminated. It is this use of WGS that may have the biggest impact on food safety and public health in the future by significantly reducing the number of contaminated products from entering the market.

As more conclusive WGS information becomes available on the diversity of bacterial traits that are related to adaptation, survival, competitive ability, and metabolic preferences, we anticipate that the application of WGS will expand from the current focus on pathogens to product spoilage and shelf life. An understanding of bacterial genes that impact public health, as well as those that are associated with product spoilage, will help the industry produce foods that are safer and superior in shelf life and quality.

However, some factors are limiting the implementation of WGS by the industry. These include the cost of implementing and using the technology, which still is prohibitive for many companies, and the uncertainty in the frequency with which this high precision technology will be applicable in their own microbiological control systems.

### Culture-independent diagnostics and metagenomics

WGS can provide all the information about a bacterial genotype and its relationship with the closely related strains; however, it is dependent on the availability of an isolate to characterize. While the FDA and FSIS must recover an isolate and characterize it for any regulatory consideration, in the medical arena, these developments are driven by the need to provide diagnostic, actionable information as fast as possible to guide patient therapy. With culture-independent tests (CIDTs), such information may be generated in hours as opposed to days with traditional culture. For this reason, an increasing number of clinical laboratories are implementing CIDTs. These include commercial multianalyte polymerase chain reaction panels and enzyme immune assays that can be applied directly to a patient sample without the need for culturing. While this approach may provide expeditious results and detect the suspect causative agents without a culture, it is also threatening the ability of public health to detect foodborne outbreaks because no isolate is available for subtyping (Marder *et al.*, 2017).

The CDC, FDA, and FSIS are working with their state partners and the Association of Public Health Laboratories (APHL) to address this problem. The long-term solution is to develop metagenomics methods that are independent of culture and combine both the detection and subtyping of the infecting organisms directly from the specimen. Two approaches are being pursued: amplicon sequencing and random shotgun sequencing. In the former, genetic targets that are specific to the pathogens identified (e.g., virulence genes and/or core genes as used in the cgMLST schemas) are amplified and sequenced, making them compatible with current MLST strain subtyping methods. This approach can be pursued using existing technology and may therefore be implemented within a few years.

In the latter approach, all genetic material in the specimen is sequenced without previous knowledge about the etiologic agent. The sequence data undergo extensive bioinformatics analysis, enabling a direct phylogenetic comparison of all microbes in the specimen. This approach shows promise after being used on historical specimens related to the German *E. coli* O104 outbreak of hemolytic uremic syndrome in 2011 (Loman *et al.*, 2013), and two outbreaks of *Salmonella* serotype Heidelberg in the United States (Huang *et al.*, 2017). However, both the sequencing technology and the bioinformatics have not yet matured to a level where this technology is feasible for real-time diagnostics and surveillance. Moreover, the presence of host DNA will pose an ethical issue if analyzed since these host sequences are signatures of the patient.

In food, naturally occurring microflora appear to be potent inhibitors or competitors and often stifle *in situ* genomic mining for *Salmonella*, *Listeria*, and other pathogens. To solve this problem, current metagenomics approaches rely on a preliminary cultural pre-enrichment or enrichment to allow for recovery of the target pathogen before metagenomics scanning for it. This has been demonstrated effectively in both ice cream, naturally contaminated with *L. monocytogenes*, and papayas contaminated with multiple *Salmonella* serovars (Bell *et al.*, 2016; Ottesen *et al.*, 2016). While shotgun metagenomics clearly holds great promise for culture-independent characterization of bacterial pathogens directly from environmental samples, substantial increases in sensitivity of the technology, as well as continued cost reductions for WGS, will be required before any significant shift toward metagenomics analysis in a regulatory or public health setting.

## Conclusion

WGS technology is extremely powerful and may provide nearly all the information contained in an entire bacterial genome, in both an increasingly timely and cost-efficient manner. It is a tool that is highly capable of inferring sources of contamination as well as contributing to food safety officials' ability to detect, resolve, and even prevent foodborne outbreaks with much greater precision and speed when compared to any other method used to date.

To facilitate this, the FDA, FSIS, and CDC with their partners upload raw sequence data and minimal metadata to the GenomeTrakr database at NCBI. This represents a trove of sequence data available to the entire scientific community and reflects the true investigative strength of a balanced genomic approach—one that combines the genomes of numerous food and environmental isolates with the actual isolates responsible for causing illness and outbreak events.

Federal partners are engaged in exploring how this wealth of information that WGS generates can be effectively harnessed in routine inspections and intervention processes, so product contamination events and foodborne illness can be effectively prevented. However, since potentially contaminated food is increasingly traded across borders, it is critical all regulatory, public health, industry, and scientific partners in food safety at the global level implement WGS and share their sequence data with the global community to detect and respond efficiently to international outbreaks. Despite aggressive promotion by GenomeTrakr and PulseNet International, there is a still a long way to go to reach this goal.

We anticipate that the next phase of WGS applications and metagenomics will further improve outbreak investigations, including our ability to attribute sporadic illness to specific food categories. Our knowledge about specific genes that may be associated with virulence, pathogenicity, survival, adaptability, antimicrobial and biocide/antiseptic resistance, food quality and spoilage, and dissemination of such genes within microbial communities will further strengthen not only the preventative and control aspects of food safety and public health but will also help to improve the quality and shelf life of foods.
